# Effects of Temperature Treatments on Cytosine-Methylation Profiles of Diploid and Autotetraploid Plants of the Alpine Species *Ranunculus kuepferi* (Ranunculaceae)

**DOI:** 10.3389/fpls.2020.00435

**Published:** 2020-04-08

**Authors:** Eleni Syngelaki, Christoph C. F. Schinkel, Simone Klatt, Elvira Hörandl

**Affiliations:** ^1^ Department of Systematics, Biodiversity and Evolution of Plants (with Herbarium), Albrecht-von-Haller-Institute for Plant Sciences, Georg-August-Universität Göttingen, Göttingen, Germany; ^2^ Section Safety and Environmental Protection, Georg-August-Universität Göttingen, Göttingen, Germany

**Keywords:** abiotic stress, alpine plants, apomixis, DNA methylation, methylation-sensitive amplified fragment length polymorphisms, polyploidy, *Ranunculus kuepferi*

## Abstract

The exposure to environmental stress can trigger epigenetic variation, which may have several evolutionary consequences. Polyploidy seems to affect the DNA methylation profiles. Nevertheless, it abides unclear whether temperature stress can induce methylations changes in different cytotypes and to what extent a treatment shift is translated to an epigenetic response. A suitable model system for studying these questions is *Ranunculus kuepferi*, an alpine perennial herb. Diploid and autotetraploid individuals of *R. kuepferi* were exposed to cold (+7°C day/+2°C night; frost treatment −1°C cold shocks for 3 nights per week) and warm (+15° day/+10°C night) conditions in climate growth chambers for two consecutive flowering periods and shifted from one condition to the other after the first flowering period. Methylation-sensitive amplified fragment-length polymorphism markers were applied for both years, to track down possible alterations induced by the stress treatments. Patterns of methylation suggested that cytotypes differed significantly in their profiles, independent from year of treatment. Likewise, the treatment shift had an impact on both cytotypes, resulting in significantly less epiloci, regardless the shift's direction. The AMOVAs revealed higher variation within than among treatments in diploids. In tetraploids, internally-methylated loci had a higher variation among than within treatments, as a response to temperature's change in both directions, and support the hypothesis of temperature stress affecting the epigenetic variation. Results suggest that the temperature-sensitivity of DNA methylation patterns shows a highly dynamic phenotypic plasticity in *R. kuepferi*, as both cytotypes responded to temperature shifts. Furthermore, ploidy level, even without effects of hybridization, has an important effect on epigenetic background variation, which may be correlated with the DNA methylation dynamics during cold acclimation.

## Introduction

Epigenetic studies try to explain the heritable changes in gene expression and function which determine the phenotype of an organism and cannot be linked to DNA sequence changes (Richards, 2006). There are mainly four different epigenetic mechanisms: a) methylation of cytosine residues in the DNA, b) remodeling of chromatin structure through chemical modification of histone proteins, c) histone proteins modification that can lead to extent alteration of DNA wrapping, and d) regulatory processes mediated by small and non-coding RNA molecules (miRNA). These processes do not act independently from each other and can produce epigenetic changes that affect genes expression, by e.g. activating, reducing or completely disabling the activity of particular genes (Berger, 2007).

DNA methylation is defined as the addiction, catalyzed by several methyltransferases, of a methyl group to the 5' C of a cytosine residue in the DNA sequence, which is associated with silencing of transposons, imprinting, and silencing of both transgenes and endogenous genes (e.g. Grossniklaus et al., 2001; Zilberman et al., 2007; Jones, 2012). In plants, DNA methylation is the best understood epigenetic mechanism and several studies intimate it exhibits a transgenerational inheritance (e.g. Vaughn et al., 2007; Johannes et al., 2009; Finnegan, 2010; Hirsch et al., 2013). It is well documented that DNA methylation can be dynamic, as biotic or abiotic environmental stimuli can trigger methylation changes and lead to different DNA methylation profiles (e.g. Sherman and Talbert, 2002; Dowen et al., 2012). Except for environmental stimuli, genomic stresses such as hybridization and polyploidization can induce DNA methylation changes (Adams and Wendel, 2005; Grant-Downton and Dickinson, 2005; Dong et al., 2006; Jones, 2012). Furthermore, DNA methylation has been exhibited to mediate phenotypic plasticity within a single generation (Bossdorf et al., 2010) and between generations (Boyko et al., 2010).

Polyploidy is a heritable condition of organisms that refers to the possession of more than two complete sets of chromosomes and is considered to have evolutionary consequences on angiosperms (Comai, 2005), as polyploidization events in plants seem to be correlated with phenotypic innovation and speciation (Madlung, 2013; Soltis et al., 2014). During the formation of polyploids, several alterations in DNA methylation in allo- and autopolyploids are occurring (Li et al., 2011). As DNA methylation is related with regulation of gene expression (Bird, 2002; Yan et al., 2010; Law and Jacobsen, 2010), it is implied the decisive role of epigenetic regulation in regaining the genomic balance and the structural and functional remodeling after polyploidization (Soltis et al., 2010; Hegarty et al., 2011; Madlung and Wendel, 2013; Alonso et al., 2016b). Several studies demonstrate that the epigenetic consequences of polyploidy can lead to gene expression alterations (e.g. gene silencing) and genome-wide transcriptional rewiring (Baubec et al., 2010; Li et al., 2011; Song and Chen, 2016). However, most studies deal with polyploid hybrids (allopolyploids). Allopolyploids show more pronounced alterations in gene expression than autopolyploids due to effects of a hybrid genome additionally to genome duplication (e.g., Chen, 2007). Moreover, DNA methylation alterations can be ignited *ad hoc* by polyploidization during the first generations following the event (Paun et al., 2007). A variation in the patterns of cytosine methylation regarding wild populations of polyploids putatively unravels their local adaptation and functional plasticity (Paun et al., 2010; Rois et al., 2013; Schulz et al., 2014) and may be advantageous for the invasion success of some species (Ainouche et al., 2009).

Uncovering the putative role that polyploidy plays at adaptation to extreme conditions is correlated with the several effects of polyploidy on vigor, physiology, morphology, and other adaptive traits (Li et al., 2011; te Beest et al., 2012). Successful polyploidization events can result in increased survival fitness in harsher environments and may have important side effects on mechanisms, which are related to stress response (Li et al., 2011). Hence, it is hypothesized that polyploidy helps plants to adjust their growth and exposure of reproductive tissues to cold temperatures by offering a putative adaptive advantage in alpine dwarfism (Körner, 2003), as polyploids achieve it by reducing cell number and increasing cell size (te Beest et al., 2012). This strategy can additionally help for rapid sprouting of the polyploidy plants directly after snow melting (Körner, 2003). According to Comai (2005), a selective advantage of polyploidy can be the way it may affect the mode of reproduction of the organism in absence of sexual mates, by favoring the establishment of asexual reproduction. The perpetuation of asexual reproduction mode on some polyploids is suggested that profits further their adaptation under stress conditions (Körner, 2003).

A suitable model system for studying the correlation of temperature effects, ploidy level, and methylation profiles is *Ranunculus kuepferi* Greuter and Burdet, a high-mountain perennial herb. The species occurs mainly with diploid and autotetraploid cytotypes and has a gradient distribution primarily across the European Alps and at altitudes between 1300 and 2800 m (Burnier et al., 2009; Cosendai and Hörandl, 2010; Cosendai et al., 2011; Kirchheimer et al., 2016; Schinkel et al., 2016). The comparison of diploid and autotetraploid cytotypes facilitates the study of effects of genome duplication without side-effects of hybridity. Regarding the reproduction mode of these cytotypes, diploid plants are predominantly sexual, whereas tetraploid plants are facultative apomictic (aposporous), with varying proportions of sexual and asexual seeds (Burnier et al., 2009; Schinkel et al., 2016).

Through the distribution of the species a pronounced geographical parthenogenesis in the European Alps (Cosendai et al., 2013) is indicated: diploid populations are restricted to the south-western Alps, while tetraploid populations colonize previously glaciated areas in the northern, central, and eastern Alps (Küpfer, 1974; Burnier et al., 2009; Cosendai and Hörandl, 2010) as well as the northern Apennines and Corsica. Tetraploid populations arise also at higher elevations in the European Alps than diploids and exhibit a pronounced niche shift towards colder temperatures (Kirchheimer et al., 2016; Schinkel et al., 2016). This niche differentiation between diploid and tetraploid cytotypes of *R. kuepferi* is associated with their reproduction mode, and asexual taxa seem to have a distributional advantage (Kirchheimer et al., 2018).

Previous population genetic studies using AFLPs throughout the range of the species revealed that sexual and apomictic populations show a very low genetic divergence, as only 3% of AFLP fragments were specific for tetraploids, while all others were shared with the diploid cytotype (Cosendai et al., 2011). Genetic differentiation and diversity measures within cytotypes were on a similar level (e.g., Fsts are around 0.3 for both cytotypes; Cosendai et al., 2013). Only diploids showed geographical structure in their refugial areas, comprising six genetic partitions, whereas tetraploid populations exhibited no geographical structure and comprised just three genetic partitions that were derived from the diploids' gene pool (Cosendai et al., 2013). Moreover, a high individual genetic diversity in tetraploids is observed and is in line with multiple origins, allelic diversity and high frequencies of facultative sexuality (Cosendai et al., 2013). A molecular dating revealed that the tetraploid cytotype originated only 10–80 kyears ago (Kirchheimer et al., 2018), probably by multiple and recurrent autopolyploidization events (Cosendai et al., 2011; Schinkel et al., 2017). Previous studies on methylation variation in natural populations suggested pronounced differences between cytotypes, and correlations of methylation variation to climatic gradients according to elevation, but not to spatial distribution (Schinkel et al. *subm*.). Hence, we hypothesize that the niche shift of tetraploids in the Alps has rather an epigenetic than a genetic background. However, in natural populations it is difficult to entangle various environmental factors, and to discriminate between phenotypic plasticity and heritable traits.

Herein, we employed the two main cytotypes of the perennial species *R. kuepferi* and we exposed plants collected in the Alps under different temperature treatments under controlled conditions, to appraise the putative DNA methylation changes. By assessing methylation variation with methylation-sensitive amplified fragment length polymorphisms (MS-AFLPs) we focus on entangling whether the methylation profiles of vegetative parts differentiate according to ploidy levels, and how the cytotypes are affected by a change in cold/warm conditions. We hypothesize a temperature-sensitivity of methylation patterns, as a potential to respond rapidly to stressful environments. We would expect that cytotypes respond differentially to stress treatments. Since we analyze here the same individuals under different treatments in consecutive years, we test here mainly for phenotypic plasticity of perennial plants. By differential analysis of types of epiloci we further try to get insights into the dynamics of epigenetic change. A detailed study on correlations of mode of reproduction and methylations will be presented elsewhere.

## Materials and Methods

### Plant Material and Experimental Design

*Ranunculus kuepferi* plants, representing diploid and tetraploid cytotypes were collected at 81 sampling sites throughout the distribution range of the species in the European Alps (Kirchheimer et al., 2016) during the growing seasons of 2013 and 2014. These plants were re-potted in garden soil at the Old Botanical Garden of Göttingen University and overwintered outdoors, while their ploidy level was defined *via* flow cytometry measurements of silica gel dried leaf material collected in the field (Schinkel et al., 2016). To investigate the implied temperature preferences of the two cytotypes (Kirchheimer et al., 2016), an experiment based on the exposure to different temperature conditions, during the sprouting and flowering period, was designed and conducted from 2014 onwards (see Klatt et al., 2018). Hence, a subset of diploid and tetraploid individuals was placed in two climate chambers MC1000E (Snijders Scientific, Tilburg, Netherlands), where cold and warm temperature treatments were implemented, while light regime (photoperiod: 16 h; 10 h full light [700 µmol m^-2^s^-1^]) and all other parameters were kept equal. In the first chamber a cold treatment was applied (+7°C day/+2°C night; frost treatment: −1°C cold shocks for 3 nights per week), mimicking the typical, harsh high alpine temperature conditions at the habitats of the tetraploid cytotype (Schinkel et al., 2016), while in the second chamber a warm treatment was applied (+15° day/+10°C night). In the cold treatment, the repeated moderate frost treatment is simulating temperature conditions occurring in high mountains and provokes frost injury in reproductive shoots, which subsequently could emerge in full fruit loss (Ladinig et al., 2013); similar damaging effects were observed by Klatt et al. (2018).

To elucidate further the effects of temperature treatments on cytotypes, a reciprocal test, by rotating the cold treated plants to the warm and *vice versa*, was performed. In spring of 2016 (third consecutive flowering period under first treatment) and 2017 (first flowering period after the rotation), leaf material was collected from the plants and was stored in silica gel. The individuals were categorized into four groups regarding their ploidy: Diploids 1, Tetraploids 1, Diploids 2, and Tetraploids 2 (from now on, mentioned as D1, T1, D2, and T2, respectively). The groups that are numbered with 1 were placed first under cold treatment, while the rest of them were first placed under warm treatment. A subset of 100 individuals (25 per group; **Supplementary Data Table 1**), originated from 57 populations, was selected to proceed with the molecular analysis for both years. The sampling was randomized and was targeted to cover as precisely as possible the distribution range of the species in the Alps (see map in **Supplementary Data Figure 1**).

### MS-AFLP Analysis

The DNA from the leaf material was isolated using the Qiagen DNeasy Plant Mini Kit, with a slightly modified protocol. At the second step, 360 μl AP1 Buffer and 40 μl PVP 2.6% were added and incubation time for the elution is prolonged 30 min. The PVP was added to remove polyphenolic compounds from plant DNA extracts by forming hydrogen bonds with them, as they can deactivate proteins and hence inhibit downstream reactions e.g. PCR (Healey et al., 2014).

In order to investigate their epigenetic response, through the highlighting of the genome-wide patterns of epigenetic variation (e.g. Salmon et al., 2008; Massicotte et al., 2011; Herrera et al., 2012), methylation-sensitive amplified fragment-length polymorphisms (MS-AFLPs) were conducted. MS-AFLPs, as a methylation detecting approach, can be applied to non-model plants for which the genome has not been sequenced yet and assess cytosine methylation state in a large number of anonymous loci, which are randomly distributed over the genome (Schrey et al., 2013).

The extracted samples of 100 individuals for each treatment year were screened according to a slightly modified protocol of Paun and Schönswetter (2012). Restriction and ligation were carried out in two parallel reactions, each one with a different restriction enzyme. The restriction enzymes, which were used, are MspI & HpaII. They are methylation sensitive restriction enzymes, i.e. isoschizomers, that recognize the same DNA sequence (CCGG), but differ in the sensitivity regarding the methylation state of C, and used as the frequent cutters, while EcoRI is used as the rare cutter. Ligation products were subjected to pre-selective amplification, whereupon selective amplification was performed with a set of three primer combinations with three selective nucleotides to each primer, used before for an AFLP analysis on the species (Cosendai et al., 2011). Ligation, pre-selective, and selective amplification products went through a quality and quantity check on a 1.5% agarose gel and diluted 10-fold dilution prior to pre-selective, selective amplification and fragment analyses, respectively.

The final selective-PCR products were prepared with GeneScan ROX 500 (Thermo Fisher Scientific, Waltham, MA, USA) as the internal size standard and fragment analyzed on the ABI Prism 3700/3730 (Applied Biosystems, Waltham, MA, USA) capillary sequencer. An attempt to increase the precision of the final results was performed, by tracking down genotyping errors and cleaning up the data sets (Bonin et al., 2004). The technical reproducibility of resulting electropherograms was checked by replicating 100% of accessions, i.e. duplicates were produced for every sample used throughout the MSAP protocol steps, to minimize the false positive fragment peaks. Accounting for the modified lab protocol for our species to overcome the sensitivity of restriction enzymes, the guidelines of ASA for the importance of replicability and reproducibility of scientific work (Wasserstein and Lazar, 2016) and the optimization of fragment detection (Arrigo et al., 2009), the risk of false positive results was minimized.

### Fragment Scoring

The analysis of electropherograms and fragment scoring were performed using the following scoring pipeline: Peakscanner v.2 (Applied Biosystems, Life Technologies Corporation, Carlsbad, California, USA), RawGeno 2.0-1 (Arrigo et al., 2009), and MSAP_calc script (Schulz et al., 2013). We transformed electropherograms of raw data into a binary dominant-marker matrix. Peak Scanner2 was used to determine the height, width, and the area of all peaks. The output of the Peak Scanner2 was then imported to RawGeno 2.0-1 to proceed with the binning of detected peaks, the analysis of replication and the filtering of samples of low quality.

RawGeno handles a single dye color at a time, so presence/absence of fragments binary matrices were obtained for each of the three dyes (Blue; FAM, Green; HEX, Yellow; NED) and then they were merged in a final binary matrix. Fragments between 50 and 600 base pairs were scored. In order to optimize the dataset, a run of RawGeno with an R script (Arrigo et al., 2009) was performed, which checked stepwise (~5,760 steps) the binning and filtering parameters. Going through the resulting table, the optimal combination of the parameters was chosen for each dye (**Supplementary Data Table 2**) and the respective binary matrices were produced. The selection of parameters represent a balance between quality measures, e.g. the error rate and bin reproducibility, and informativity, measured with the data polymorphism.

The merged binary matrix of optimized dataset for each treatment year was dealt with MSAP_calc script in R (Schulz et al., 2013), to distinguish the four possible methylation conditions as they are described in Schulz et al., 2013, using the “Mixed Scoring 2” approach for scoring the following conditions: I) no methylation (both MspI and HpaII cut the restriction site), II) holo- or hemi-methylation of internal cytosine (^HMe^CG or ^Me^CG; MspI cuts the restriction site), III) hemimethylation of external cytosine (^HMe^CCG; HpaII cuts the restriction site), and IV) holomethylation of external cytosine or of both cytosines or hemi-methylation of both cytosines or mutations (none of them cuts the restriction site). In “Mixed Scoring 2” condition I was scored as 100 (non-methylated), condition II as 010 (internally-methylated), condition III as 001 (externally-methylated), and condition IV as “000” and refers to a non-distinguishable situation, e.g. an ambiguous methylation or a mutation status. Condition IV was, therefore, excluded from further statistical analyses. In “Mixed Scoring 2” approach both group of fragments are comprised, so potential inadequacies of methylation- and non-methylation Scoring were avoided and more of the underlying information of the epiloci is utilized (Alonso et al., 2016a).

### Statistical Analyses

MSAP_calc script returned an epigenetic binary matrix, which presents the methylation condition (externally-, internally- and non-methylated) of each epilocus with the corresponding score and a matrix with descriptive parameters at the group level. The subsequent statistical analyses were mainly performed in R (R Development Core Team, 2019) under R Studio (RStudio Team, 2016).

The descriptive parameters matrices were adopted from the MSAP_calc script and used as input for barplots in ggplot2 (Wickham, 2009). In accordance with descriptive statistics, the percentages of each epilocus for each individual were calculated regarding the predefined groups for each year of analysis and the groups with some individuals through the years. These percentages were used to produce the respective boxplots with ggplot2 R package and were arcsine-transformed in Excel 2016 to match a normal distribution of data. Based on the arcsine-transformed percentages, multiway ANOVAs regarding pairwise comparisons of the groups (ploidy *versus* treatments, year *versus* groups) and the non-parametric tests of Wilcoxon and Kruskal-Wallis were computed.

To estimate the epigenetic variances within and among the groups for each treatment year, the groups of same individuals through the treatment years, the cytotypes, and the treatments as well as the respective epigenetic phenotypic differentiation (Φ_ST_), several global and locus-by-locus AMOVAs were conducted, using ARLEQUIN (Excoffier and Lischer, 2010), version 3.5.2.2. Binary matrices were treated before with AFLPdat script (Ehrich, 2006) to get an output file in Arlequin format. Both modes of AMOVA were based on the option for haplotypic data computed with pairwise differences, a gamma value of 0 and a permutation number of 50.000. Measures as F_ST_ (homologous to Φ_ST_), which describe genetic population structuring, should be equally applied to specify population or group differentiation at the epigenetic level (Bossdorf et al., 2008).

## Results

### Epigenetic Patterns of Each Year of Treatment

The MS-AFLP analysis was conducted on all (100) individuals for the 2017 treatment and on 99 of them for the 2016 treatment, as RawGeno scoring discarded one individual belonging to T2, because of its low quality. Across the 2016 analysis, 754 fragments were scored, while for 2017, the scorable fragments were 493. Furthermore, in the D1, T1, D2, and T2 groups 401, 562, 485, 559 and 260, 258, 266, 232 polymorphic markers were detected for 2016 and 2017 analyses, respectively (see **Table 1**).

**Table 1:** Measures of epigenetic diversity within the four groups of *Ranunculus kuepferi* obtained for all the types of epiloci.

	Diploids 1 (D1)	Tetraploids 1 (T1)	Diploids 2 (D2)	Tetraploids 2 (T2)
	**2016**			
**All (754 epiloci)**				
Polymorphic epiloci (%)	53.18	74.54	64.32	74.14
Private epiloci	32	43	32	44
Mean Shannon's diversity	0.27	0.45	0.36	0.44
**Externally-methylated (255 epiloci)**				
Polymorphic epiloci (%)	45.1	65.49	60.39	60.39
Private epiloci	19	23	22	13
Mean Shannon's diversity	0.17	0.38	0.27	0.31
**Internally-methylated (254 epiloci)**				
Polymorphic epiloci (%)	72.05	67.72	74.41	74.02
Private epiloci	12	7	8	22
Mean Shannon's diversity	0.42	0.43	0.47	0.44
**Non-methylated (245 epiloci)**				
Polymorphic epiloci (%)	42.04	91.02	57.96	88.57
Private epiloci	1	13	2	9
Mean Shannon's diversity	0.21	0.55	0.34	0.57
****	**2017**			
**All (493 epiloci)**				
Polymorphic epiloci (%)	52.74	52.33	53.96	47.06
Private epiloci	52	41	96	27
Mean Shannon's diversity	0.21	0.23	0.23	0.23
**Externally-methylated (287 epiloci)**				
Polymorphic epiloci (%)	55.05	65.51	54.7	58.89
Private epiloci	18	36	26	16
Mean Shannon's diversity	0.22	0.29	0.25	0.32
**Internally-methylated (119 epiloci)**				
Polymorphic epiloci (%)	48.74	26.89	50.42	30.25
Private epiloci	26	2	38	11
Mean Shannon's diversity	0.17	0.1	0.2	0.12
**Non-methylated (87 epiloci)**				
Polymorphic epiloci (%)	50.57	43.68	56.32	31.03
Private epiloci	8	3	32	0
Mean Shannon's diversity	0.23	0.19	0.2	0.09

In 2016, all diversity measures (percentage of polymorphic loci, private loci, and Shannon index) suggest an overall higher diversity for tetraploids, mostly due to internally-methylated and non-methylated markers. In 2017, this trait is reversed as diploids show a higher diversity of markers in these epiloci. All the described differences were consistent under cold treatment and more sporadic under warm treatment (**Table 1**).

**Figure 1:**
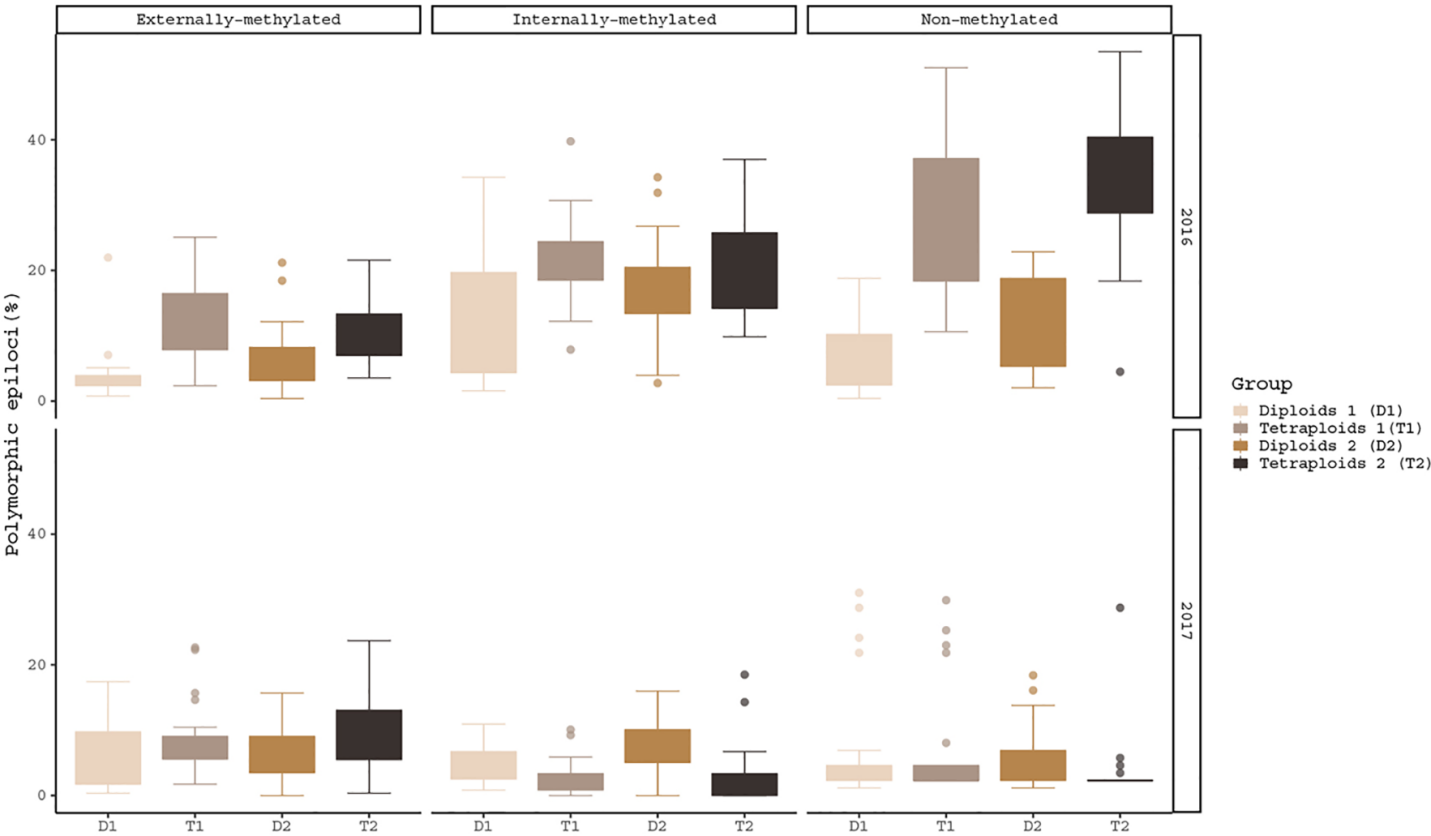
Boxplots of polymorphic epiloci (%) of the four *R. kuepferi* groups for each year of treatment. For test statistics see **Table S3**.

The hypothesis of the ploidy effect is confirmed for both years of analysis, as the three different types of epiloci for diploids and tetraploids differed significantly under the same conditions (**Figure 1**). More specifically, in externally-, non- and internally-methylated epiloci the ploidy levels under cold treatment (pairs: T1/D1 for 2016 and T2/D2 for 2017) differ significantly (p-values***_2016_*** < 0.001, < 0.001, < 0.05 and p-values***_2017_*** < 0.05, < 0.01, < 0.001 respectively). The ploidy effect is also observed under warm treatment for the non-methylated epiloci of 2016 analysis and externally- as well the internally-methylated epiloci of 2017 analysis (p-value***_2016_*** = < 0.001 and p-values***_2017_*** = 0.0373, 0.04599 respectively). A treatment effect within the same year and ploidy level is prominent only for the internally-methylated epiloci of the diploid individuals under 2017 analysis (p-value***_2017_*** < 0.05).

### Correlation of Epigenetic Patterns Under the Treatment Shift

**Figure 2:**
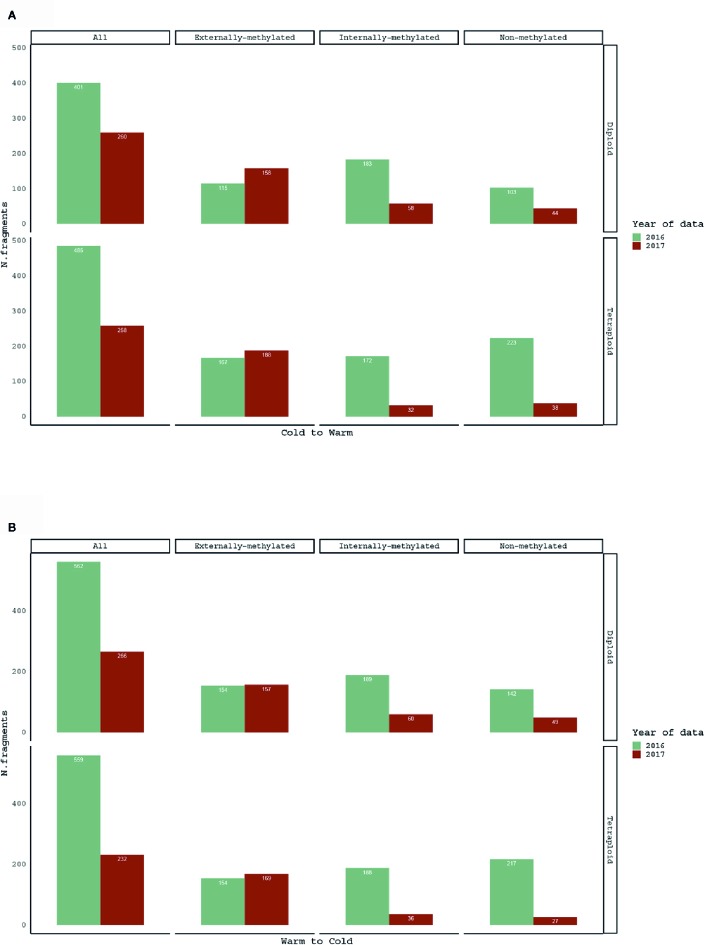
Barplots for comparison of scorable fragments, in absolute numbers (see data in **Table 1**), for **(A)** Cold to Warm and **(B)** Warm to Cold (*vice versa*) shift, among the same *R. kuepferi* individuals.

In order to test how the methylation patterns change with the reversed treatment, pairwise comparisons of same individuals between 2016 and 2017 treatment years were performed. The treatment shift, as for both directions (Warm to Cold and Cold to Warm), affected the number and the proportion of polymorphic epiloci for the groups with same individuals (**Figure 2**).

**Figure 3:**
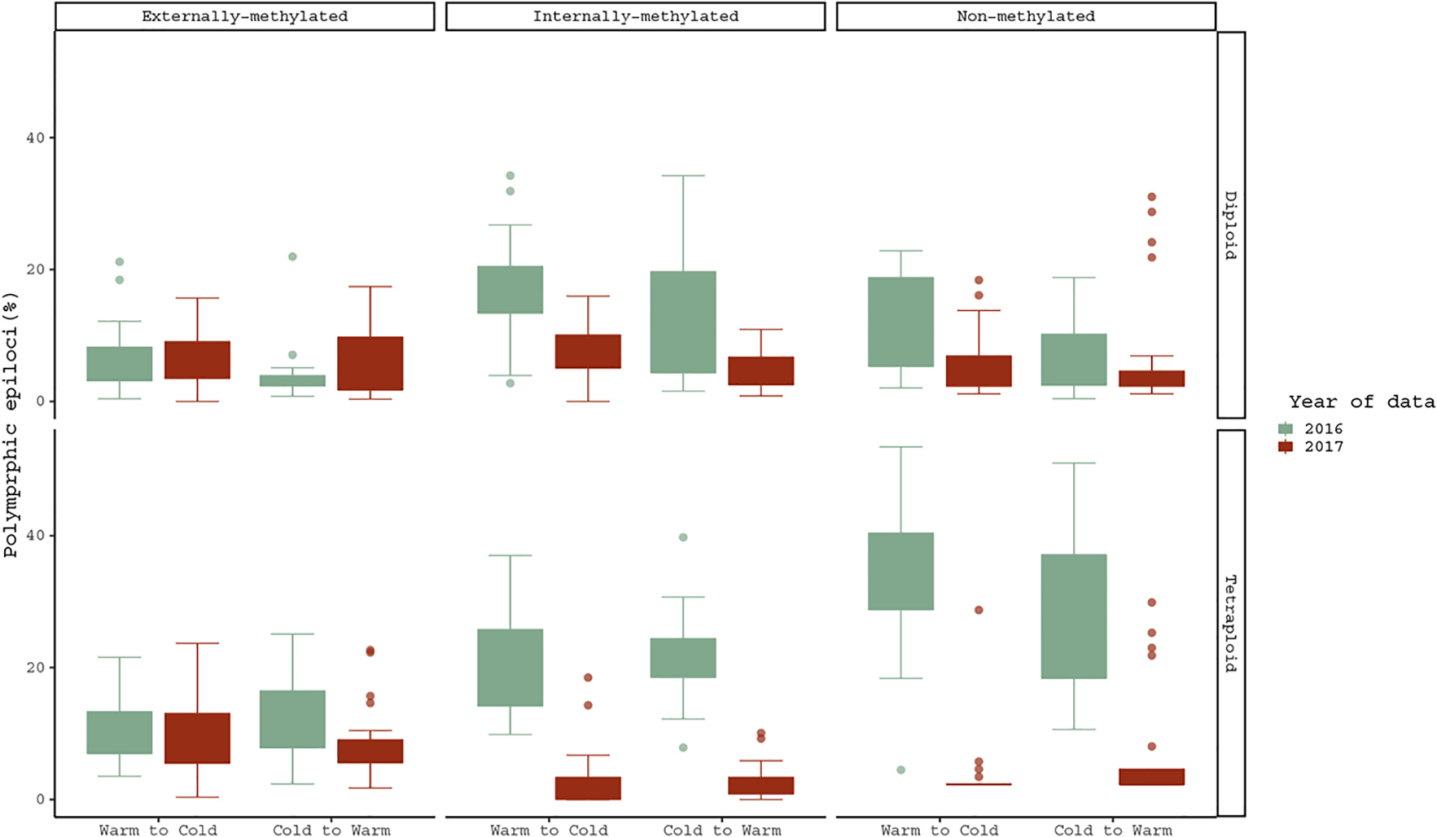
Pairwise comparison of polymorphic epiloci (%) regarding the same *R. kuepferi* individuals, which went through the reciprocal treatment shift from 2016 to 2017. For test statistics see **Table S3**.

Regarding the treatment effect hypothesis through descriptive statistics, boxplots and statistical significant tests propose that both cytotypes changed significantly their methylations under Cold to Warm shift and vice versa, for non- and internally-methylated epiloci (**Figure 3**).

However, the cytotypes do not differ from each other in their response to the temperature changes. Furthermore, in non- and internally-methylated epiloci the pair D1/D2 differs significantly (p-values <0.001 from Warm to Cold and p-values <0.005, < 0.001 from Cold to Warm), and as well does the T1/T2 pair (p-values <0.001 for both directions). Moreover, the corresponding comparison of tetraploids under the shift from Cold to Warm treatment gave significant differences also for the case of externally-methylated epiloci (p-value = < 0.005).

Concerning the global AMOVA results, a higher epigenetic variation within than among the groups was found in the diploids for all types of epiloci. Regarding the groups of tetraploids, the epigenetic variation is greater among than within them for non- and internally-methylated epiloci, but not for externally-methylated ones (**Table 2**). These differences in variation were accounted for both directions of treatment shifts. The Φ_ST_ values are greater than 0.15, except for the externally-methylated epiloci of diploids that were alternated from Cold to Warm treatment. Values are ascending from externally- to non- and internally-methylated epiloci.

**Table 2:** Analysis of Molecular Variance (AMOVA) of epiloci, overall and for each type, under both directions of treatment's shift, for the groups of the same individuals of *Ranunculus kuepferi*. 2x: Diploid, 4x: Tetraploid.

Epiloci	Overall				Externally-methylated				Internally-methylated				Non-methylated			
Shift's direction	Warm to Cold		Cold to Warm		Warm to Cold		Cold to Warm		Warm to Cold		Cold to Warm		Warm to Cold		Cold to Warm	
Ploidy level	2×	4×	2×	4×	2×	4×	2×	4×	2×	4×	2×	4×	2×	4×	2×	4×
**AMOVA global**																
Variation among groups (%)	33.38	40.08	31.61	36.01	10.26	22.78	19.28	15.3	48.22	55.93	31.61	57.8	45	57.18	49.54	51.68
Variation within groups (%)	66.62	59.92	68.39	63.99	89.74	77.22	80.72	84.7	51.78	44.07	68.39	42.2	55	42.82	50.46	48.32
Φ_ST_	0.33	0.4	0.32	0.36	0.1	0.23	0.19	0.15	0.48	0.56	0.32	0.58	0.45	0.57	0.5	0.52
P-value	<0.001	<0.001	<0.001	< 0.001	<0.001	<0.001	<0.001	<0.001	<0.001	< 0.001	<0.001	<0.001	<0.001	<0.001	<0.001	<0.001
**AMOVA locus-by-locus**																
Significantly differentiated loci	30	41	24	38	7	19	8	18	16	10	8	12	6	12	6	9
Significantly differentiated loci (%)	30.93	38.68	25	34.86	12.96	30.64	15.38	27.69	66.66	50	33.33	60	31.58	50	30	37.5

Similarly, locus-by-locus AMOVA revealed lowest percentages of significantly differentiated epiloci in externally-methylated epiloci, followed by non-methylated epiloci, and highest percentages in internally-methylated epiloci, for both ploidy levels and treatment shifts. In addition, under the change from Warm to Cold both ploidy levels exhibit more significantly differentiated epiloci, than the ones for the reciprocal change. For this tendency we observed the exceptions of externally- and internally-methylated epiloci for diploids and tetraploids individuals, respectively (**Table 2**).

## Discussion

In the current study, patterns of epigenetic variation in two cytotypes of *R. kuepferi* along cold (stress) and warm (control) temperature treatments were explored and comparisons for the same individuals after the shift of treatments were performed. The results confirmed the correlation of methylation profiles with the ploidy level, under the same treatment conditions and for both years of analyses. Furthermore, tetraploids had significantly more epiloci than diploids, regarding overall, externally- and non-methylated epiloci under cold treatment of 2016, while for the 2017 analyses the diploid individuals, have more internally-methylated epiloci under both treatments, more non-methylated epiloci under cold treatment and less externally-methylated epiloci under both treatments.

The response to the reciprocal change of treatments is supported by the comparisons of methylation patterns of same individuals before and after the treatment shift. Overall, the treatment shift resulted in a drastic decrease of scorable fragments and, regardless of the shift's direction, a significantly lower number of internally- and non-methylated epiloci. Moreover, the significant higher number of internally-methylated epiloci under cold treatment, compared to warm in the same year, indicates a temperature-sensitivity of DNA methylation patterns of 2017 among diploids.

Subsequently, we will attempt to interpret all results according to the two factors ploidy level and treatment.

### Epigenetic Patterns and Ploidy Level

The observed difference in methylation patterns of the ploidy levels, for both years of analysis, is in contrast to the very low genetic differentiation of cytotypes in wild populations in the AFLP study of Cosendai et al. (2011, 2013). Moreover, variation in DNA methylation patterns over short timescales appears to be spontaneous and independent from genetic background (Vidalis et al., 2016). The epigenetic differentiation of cytotypes was also observed in methylation patterns studied in wild populations of *R. kuepferi* (Schinkel et al., *subm.*). Since tetraploid *R. kuepferi* is autopolyploid, we can also rule out side-effects of hybridity on methylation patterns (e.g., Chen, 2007). The consistency of results suggests that polyploidization affects methylation variation independently from genetic background variation and environment. Strikingly, for 2016, tetraploids showed significantly more epiloci than diploids do. Other studies have shown that autopolyploidy can trigger *de novo* methylation in the model system *Taraxacum officinale* (Verhoeven et al., 2010a) and the endemic species of the Iberian Peninsula, *Dianthus broteri* (Alonso et al., 2016b) as well as increase the global cytosine methylation in six species of the grass species complex Cymbopogon (Lavania et al., 2012).

Cold treatment conditions were quite similar to the natural habitat conditions of the tetraploid *R. kuepferi* plants in the Alps (see also Klatt et al., 2018). They have an extended biogeographical and altitudinal distribution (Cosendai and Hörandl, 2010; Schinkel et al., 2016; Kirchheimer et al., 2016), but neither geographical structure nor isolation-by-distance appeared in their gene pool (Cosendai et al., 2013). The lack of genetic structure in tetraploids is probably due to their recent origin and rapid postglacial colonization of the Alps (Kirchheimer et al., 2018). Kirchheimer et al. (2016) suggested that tetraploids' niche optimum is placed in the direction of cooler conditions than the one for diploid cytotype, probably due to the change in the reproductive system of an originally warm-adapted species during postglacial re-colonization of higher regions in the Alps (Kearney, 2005; Hörandl, 2006). Tolerance to cooler conditions allows tetraploids to surmount high elevation barriers and establish new populations throughout a greater distribution range (Kirchheimer et al., 2018). The shift to facultative apomixis may have additionally facilitated rapid colonization (Kirchheimer et al., 2018). Warm treatments rather reflected the natural temperature conditions of the diploids in the southwestern Alps. DNA methylation patterns in the wild populations indeed followed rather a temperature gradient than the biogeographical pattern and they seem to correlate with reproduction mode of each cytotype (Schinkel et al., *subm.*). Previous cold/warm treatments by Klatt et al. (2018) in climate growth chambers suggested indeed some influence of temperature on mode of reproduction in diploids, and overall a high phenotypic plasticity in this trait. However, this earlier study did not include a reciprocal change of treatments. The potential correlations of reproduction mode and the epigenetic patterns of each cytotype regarding our experiments will be discussed elsewhere, as our main aim of the current study is to decipher the ploidy-treatment linked effects.

Hence, the results of the first treatment in 2016 may still reflect to a great extent the background methylation profiles from the natural habitats, potentially related to differential cold acclimation of diploids and tetraploids. Correlating these background studies with the dynamics of DNA methylation during cold acclimation (Liu et al., 2017), the findings of 2016, which represented the 3^rd^ year (2014–2016) of cultivation in growth chambers under the same conditions, are slightly deciphered.

The evolutionary aspects of stress-induced epigenetic variation as well as the epigenetic inheritance have been noteworthy discussed (e.g., Wendel and Rapp, 2005; Richards, 2006; Bossdorf et al., 2008; Richards et al., 2017). The exposure to biotic or abiotic environmental stresses can trigger epigenetic changes that seem to persist even after the stress is relieved, resulting in a stress memory that can be stable throughout the lifetime of an organism or even across generations, especially in plants (Richards et al., 2012). The observed cytotype differentiation of methylation in *R. kuepferi* could reflect such a heritable pattern as ploidy levels, which are highly stable within natural populations (Cosendai and Hörandl, 2010; Schinkel et al., 2016).

### Epigenetic Patterns and Treatment

Regarding the treatment effect on the DNA methylation patterns, only after the reciprocal change of treatments for the same individuals we observed significant changes of the methylation profiles. We suggest that an extreme change of temperature is needed to alter methylation patterns independently from ploidy variation. However, such extreme temperature shifts do occur under natural weather conditions in the Alps, and alpine plants have to be tolerant to temperature extremes between day and night, extreme low temperatures down to −24°C in higher altitudes, as well as different weather conditions and seasons (Körner, 2003). The high phenotypic plasticity in methylation patterns, which we observed in *R. kuepferi*, may be responsive to the fluctuating climatic conditions. Several studies insinuate increases in epigenetic variation in response to different environmental factors (Verhoeven et al., 2010b; Dowen et al., 2012; Verhoeven and Preite, 2013; Nicotra et al., 2015; Foust et al., 2016). The 2017 results for *R. kuepferi* may empower the argument of cold-induced DNA methylation changes, as described by e.g. Song et al. (2015) regarding the alpine to subnivale species *Chorispora bungeana*. Furthermore, in the alpine species *Wahlenbergia ceracea*, adaptive plasticity in methylation was observed in low-elevation plants (Nicotra et al., 2015), while forest trees set off several epigenetic mechanisms, including DNA methylation, to elicit rapid phenotypic variations, which help them respond to environmental changes (Mamadou et al., 2018). We cannot rule out that perennial plants like *R. kuepferi* change their methylation profile over their lifetime independently from environmental influence. However, it is unlikely that such a drastic shift from one year to another would be just an effect of ageing, as it occurred synchronously in plants from different origins. Our plants showed no signs of senescence.

Hereby, concerning the comparison of same individuals for the two years of analysis, less fragments were detected after the shift of treatments, i.e. 754 for 2016 in contrast to 493 for 2017. The “*Mixed Scoring 2*” approach used in the current study for fragment detection (Schulz et al., 2013) does score “000” for the condition IV, which refers to the uninformative state of fragments' absence due to methylation polymorphisms (Zhang et al., 2007) or restriction site polymorphisms/mutations. Methylation polymorphisms may affect both the external cytosine (e.g., ^Me^CCGG, ^Me^C^Me^CGG) as well as the internal cytosine (e.g. ^HMe^C^HMe^CGG, ^Me^C^Me^CGG), and correct interpretation of changes of methylation status from one to the other is often complicated (Fulnecek and Kovarik, 2014). Remarkably, the loss of epiloci appeared in both cytotypes of *R. kuepferi* and just affected internally-methylated and non-methylated loci. This observation may point at an inability of MspI to cut within the CG context. Hence, we cannot readily interpret our pattern as demethylation process, but rather as indicative of a high methylation dynamics, or even mutational change since 5-methylcytosin can convert to a thymine *via* deamination. However, our results suggest that types of epiloci react differentially to abiotic stress, which is in line with involvement of differential methyltransferase families and positions in the genome (e.g. Alonso et al., 2016a).

Our speculations refer to the potential of DNA methylation mechanisms to respond to abiotic environmental stress (Richards et al., 2017) and the complex network of them with the other epigenetic mechanisms. It is known that DNA methylation, as an important epigenetic mechanism, is involved in diverse biological processes such as transposon proliferation, genomic imprinting, and regulation of gene expression (e. g. Law and Jacobsen, 2010) and together with histone modifications and non-histone proteins, encompasses chromatin structure and accessibility (Zhang et al., 2018). The aforementioned phenotypic plasticity in methylations may improve the phenotypic response of plants during acclimation and adaptation to heterogeneous environments (Nicotra et al., 2010).

The lower number of internally- and non-methylated epiloci after the shift, referring to the methylation profiles of the same individuals, regardless the direction of treatment's change and the ploidy level, and the greater number of externally-methylated epiloci of tetraploid individuals, highlight likewise the response to the new abiotic conditions after the shift from cold to warm conditions. This epigenetic variation may depict the dynamics of DNA methylation under stress conditions (Bartels et al., 2018; Zhang et al., 2018) and the epigenetic control on the phenotypic plasticity of the species (Zhang et al., 2012; Richards et al., 2017).

Kooke et al. (2015) suggested that a DNA demethylation is responsible for variation of phenotypic plasticity by extending its environmental sensitivity. Furthermore, DNA methylation in response to abiotic environmental stress could regulate gene expression (e.g. Steward et al., 2002; Shan et al., 2013; Rakei et al., 2016), mostly by global demethylation of genomic DNA, while DNA methylation is believed to play a role in the maintenance of cell stability under stress (Song et al., 2015). However, since the interpretation of our methylation profiles is not straightforward we can just confer a high dynamics from our experiments.

Global AMOVA results for tetraploids reflect these DNA methylation dynamics as a response to the change of temperature, from warm to cold conditions and *vice versa*. Interestingly, regarding the shift from warm to cold, the higher variation among the tetraploid groups for the non-methylated epiloci may underline the, hypothetically, important role of methylation dynamics under cold treatment. The locus-by-locus AMOVA results, which indicate the epigenetic phenotypic differentiation at each locus, support the hypothesis of cold stress affecting the epigenetic variation, as there were overall more significantly differentiated epiloci for the shift from warm to cold, also for the diploid groups.

To summarize, the current study demonstrates a ploidy effect on the DNA methylation profiles, mainly under cold treatment, and a significant differentiation of them as a response to the reciprocal temperature treatment experiments. This phenotypic plasticity may affect the potential of the two cytotypes, and therefore also the two different reproduction modes, to tolerate cold stress. Differential response of the different types of epiloci suggest a high epimutational dynamics.

## Data Availability Statement

The datasets generated for this study can be found in Dryad https://datadryad.org/stash/share/AKNkEMAeweWRfZGjumxlDhZ0gLJS29IJp9W6UNhPOzA.

## Author Contributions

ES and EH designed research. CS, SK, and EH collected plant materials. ES, CS, and SK performed experiments. ES collected and analyzed data. ES wrote the paper with contributions from EH. All authors commented on and discussed previous versions of the paper.

## Funding

The work was supported by the German Science Foundation Deutsche Forschungsgemeinschaft DFG (project Ho4395/1-2) to EH.

## Conflict of Interest

The authors declare that the research was conducted in the absence of any commercial or financial relationships that could be construed as a potential conflict of interest.
