# Supplementary material for: Effects of Temperature Treatments on Cytosine-Methylation Profiles of Diploid and Autotetraploid Plants of the Alpine Species Ranunculus kuepferi (Ranunculaceae)
Source: Front Plant Sci. 2020 Apr 8;11:435. doi: 10.3389/fpls.2020.00435 (PMC7158262; doi:10.3389/fpls.2020.00435)
Supplement: Supplementary file 1 [file DataSheet_1.docx]

Supplementary Data

Table S1. List of individuals used for the laboratory work. Listed are the sampling ID, ploidy level, treatment, country, province and altitude (Schinkel *et al.* 2016, Klatt *et al.* 2018)

| **Sample ID** | **Leaf ploidy** | **Temperature Treatment 2016** | **Temperature Treatment 2017** | **Country** | **Region** | **Altitude** |
| --- | --- | --- | --- | --- | --- | --- |
| 3/2/3 | diploid | cold | warm | France | Provence-Alpes-Côte d'Azur | 2291 |
| 23/4/1 | diploid | cold | warm | France | Provence-Alpes-Côte d'Azur | 1616 |
| 24/4/3 | diploid | cold | warm | France | Provence-Alpes-Côte d'Azur | 1925 |
| 25/3/2 | diploid | cold | warm | France | Provence-Alpes-Côte d'Azur | 1435 |
| 26/3/3 | diploid | cold | warm | France | Provence-Alpes-Côte d'Azur | 1456 |
| 27/2/1 | diploid | cold | warm | France | Rhônes-Alpes | 1449 |
| 28/3/1 | diploid | cold | warm | Italy | Piemonte | 1685 |
| 28/3/2 | diploid | cold | warm | Italy | Piemonte | 1685 |
| 29/1/2 | diploid | cold | warm | Italy | Piemonte | 2020 |
| 30/1/3 | diploid | cold | warm | Italy | Piemonte | 1743 |
| 31/3/3 | diploid | cold | warm | Italy | Piemonte | 1937 |
| 32/4/2 | diploid | cold | warm | Italy | Piemonte | 2320 |
| 33/3/3 | diploid | cold | warm | Italy | Piemonte | 2328 |
| 112/3/1 | diploid | cold | warm | France | Provence-Alpes-Côte d'Azur | 1626 |
| 112/3/2 | diploid | cold | warm | France | Provence-Alpes-Côte d'Azur | 1626 |
| 115/3/3 | diploid | cold | warm | France | Provence-Alpes-Côte d'Azur | 1891 |
| 116/1/1 | diploid | cold | warm | France | Provence-Alpes-Côte d'Azur | 1953 |
| 116/3/3 | diploid | cold | warm | France | Provence-Alpes-Côte d'Azur | 1953 |
| 117/3/2 | diploid | cold | warm | France | Provence-Alpes-Côte d'Azur | 1632 |
| 203/4/3 | diploid | cold | warm | France | Provence-Alpes-Côte d'Azur | 1840 |
| 233/2/1 | diploid | cold | warm | France | Provence-Alpes-Côte d'Azur | 2185 |
| 28B/3/1 | diploid | cold | warm | Italy | Piemonte | 1685 |
| 31B/1/3 | diploid | cold | warm | Italy | Piemonte | 1937 |
| 31B/2/2 | diploid | cold | warm | Italy | Piemonte | 1937 |
| 32B/1/2 | diploid | cold | warm | Italy | Piemonte | 2320 |
| 17/4/3 | tetraploid | cold | warm | France | Provence-Alpes-Côte d'Azur | 2357 |
| 34/2/1 | tetraploid | cold | warm | France | Rhônes-Alpes | 2120 |
| 36/4/1 | tetraploid | cold | warm | France | Rhônes-Alpes | 2152 |
| 37/1/2 | tetraploid | cold | warm | Italy | Val d'Aosta | 2115 |
| 38/1/2 | tetraploid | cold | warm | France | Rhônes-Alpes | 2182 |
| 41/4/3 | tetraploid | cold | warm | Italy | Val d'Aosta | 2174 |
| 42/1/2 | tetraploid | cold | warm | Switzerland | Valais | 1789 |
| 47/3/3 | tetraploid | cold | warm | Switzerland | Graubunden | 2211 |
| 51/3/1 | tetraploid | cold | warm | Austria | Tirol | 2286 |
| 53/1/3 | tetraploid | cold | warm | Switzerland | Graubunden | 2456 |
| 54/2/3 | tetraploid | cold | warm | Italy | Lombardia | 2303 |
| 58/1/1 | tetraploid | cold | warm | Italy | Trentino Alto Adige/ Südtirol | 2117 |
| 58/3/3 | tetraploid | cold | warm | Italy | Trentino Alto Adige/ Südtirol | 2117 |
| 74/1/2 | tetraploid | cold | warm | Austria | Osttirol | 2117 |
| 78/3/2 | tetraploid | cold | warm | Switzerland | Valais | 2000 |
| 79/2/3 | tetraploid | cold | warm | Switzerland | Graubunden | 2280 |
| 85/4/1 | tetraploid | cold | warm | Austria | Kärnten | 2184 |
| 88/3/1 | tetraploid | cold | warm | Switzerland | Graubunden | 2300 |
| 103/4/2 | tetraploid | cold | warm | Italy | Lombardia | 2290 |
| 106/4/1 | tetraploid | cold | warm | Italy | Trentino Alto Adige/ Südtirol | 2142 |
| 111/3/2 | tetraploid | cold | warm | France | Provence-Alpes-Côte d'Azur | 2243 |
| 111/3/3 | tetraploid | cold | warm | France | Provence-Alpes-Côte d'Azur | 2243 |
| 116/4/1 | tetraploid | cold | warm | France | Provence-Alpes-Côte d'Azur | 1953 |
| 208/2/1 | tetraploid | cold | warm | France | Provence-Alpes-Côte d'Azur | 1924 |
| 3B/1/2 | tetraploid | cold | warm | France | Provence-Alpes-Côte d'Azur | 2291 |
| 3/2/2 | diploid | warm | cold | France | Provence-Alpes-Côte d'Azur | 2291 |
| 3/3/2 | diploid | warm | cold | France | Provence-Alpes-Côte d'Azur | 2291 |
| 23/3/3 | diploid | warm | cold | France | Provence-Alpes-Côte d'Azur | 1616 |
| 24/1/2 | diploid | warm | cold | France | Provence-Alpes-Côte d'Azur | 1925 |
| 25/1/3 | diploid | warm | cold | France | Provence-Alpes-Côte d'Azur | 1435 |
| 26/4/1 | diploid | warm | cold | France | Provence-Alpes-Côte d'Azur | 1456 |
| 27/2/2 | diploid | warm | cold | France | Rhônes-Alpes | 1449 |
| 28/2/3 | diploid | warm | cold | Italy | Piemonte | 1685 |
| 29/4/2 | diploid | warm | cold | Italy | Piemonte | 2020 |
| 30/1/2 | diploid | warm | cold | Italy | Piemonte | 1743 |
| 31/2/3 | diploid | warm | cold | Italy | Piemonte | 1937 |
| 32/3/3 | diploid | warm | cold | Italy | Piemonte | 2320 |
| 33/4/2 | diploid | warm | cold | Italy | Piemonte | 2328 |
| 112/1/2 | diploid | warm | cold | France | Provence-Alpes-Côte d'Azur | 1626 |
| 115/4/2 | diploid | warm | cold | France | Provence-Alpes-Côte d'Azur | 1891 |
| 116/1/3 | diploid | warm | cold | France | Provence-Alpes-Côte d'Azur | 1953 |
| 116/3/1 | diploid | warm | cold | France | Provence-Alpes-Côte d'Azur | 1953 |
| 117/3/3 | diploid | warm | cold | France | Provence-Alpes-Côte d'Azur | 1632 |
| 202/3/3 | diploid | warm | cold | France | Provence-Alpes-Côte d'Azur | 1829 |
| 203/2/2 | diploid | warm | cold | France | Provence-Alpes-Côte d'Azur | 1840 |
| 233/2/3 | diploid | warm | cold | France | Provence-Alpes-Côte d'Azur | 2185 |
| 28B/1/2 | diploid | warm | cold | Italy | Piemonte | 1685 |
| 31B/3/3 | diploid | warm | cold | Italy | Piemonte | 1937 |
| 32B/1/1 | diploid | warm | cold | Italy | Piemonte | 2320 |
| 32B/2/1 | diploid | warm | cold | Italy | Piemonte | 2320 |
| 17/4/2 | tetraploid | warm | cold | France | Provence-Alpes-Côte d'Azur | 2357 |
| 34/1/1 | tetraploid | warm | cold | France | Rhônes-Alpes | 2120 |
| 36/2/1 | tetraploid | warm | cold | France | Rhônes-Alpes | 2152 |
| 37/4/1 | tetraploid | warm | cold | Italy | Val d'Aosta | 2115 |
| 40/3/1 | tetraploid | warm | cold | Switzerland | Valais | 1860 |
| 41/2/2 | tetraploid | warm | cold | Italy | Val d'Aosta | 2174 |
| 42/3/2 | tetraploid | warm | cold | Switzerland | Valais | 1789 |
| 45/1/2 | tetraploid | warm | cold | Switzerland | Valais | 2400 |
| 47/2/2 | tetraploid | warm | cold | Switzerland | Graubunden | 2211 |
| 48/4/2 | tetraploid | warm | cold | Switzerland | Graubunden | 2262 |
| 50/2/1 | tetraploid | warm | cold | Switzerland | Graubunden | 2322 |
| 53/3/1 | tetraploid | warm | cold | Switzerland | Graubunden | 2456 |
| 54/2/2 | tetraploid | warm | cold | Italy | Lombardia | 2303 |
| 55/3/1 | tetraploid | warm | cold | Austria | Tirol | 2557 |
| 58/1/3 | tetraploid | warm | cold | Italy | Trentino Alto Adige/ Südtirol | 2117 |
| 66/2/2 | tetraploid | warm | cold | Italy | Trentino Alto Adige/ Südtirol | 2101 |
| 79/3/3 | tetraploid | warm | cold | Switzerland | Graubunden | 2280 |
| 82/3/3 | tetraploid | warm | cold | Italy | Lombardia | 2500 |
| 83/3/1 | tetraploid | warm | cold | Austria | Osttirol | 2271 |
| 84/4/2 | tetraploid | warm | cold | Austria | Kärnten | 2236 |
| 90/2/3 | tetraploid | warm | cold | Switzerland | Valais | 2477 |
| 96/2/3 | tetraploid | warm | cold | France | Provence-Alpes-Côte d'Azur | 2300 |
| 106/2/3 | tetraploid | warm | cold | Italy | Trentino Alto Adige/ Südtirol | 2142 |
| 108/3/3 | tetraploid | warm | cold | Switzerland | Graubunden | 2171 |
| 111/2/3 | tetraploid | warm | cold | France | Provence-Alpes-Côte d'Azur | 2243 |


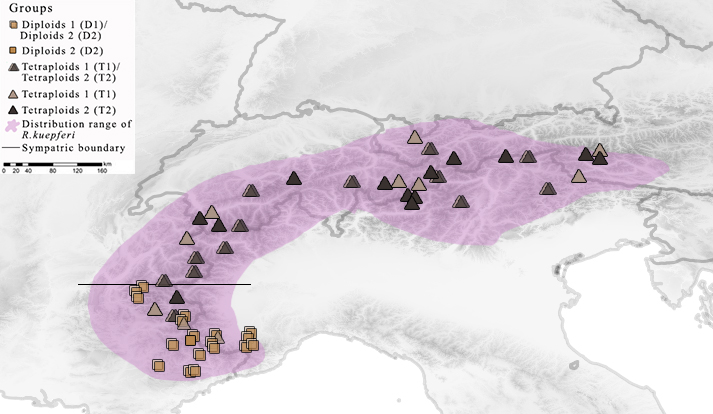


Figure S1. Distribution map of *R. kuepferi* in the Alps, indicating the populations selected for the MSAP study. Several populations were represented in both experimental groups 1 and 2 of diploid and autotetraploid cytotypes.

Table S2. The parameters, which were chosen for each dye run in RawGeno, with regard to an optimized dataset for each year. Listed are minimum and maximum bin width (bp), Relative Fluorescence Units (RFU), number of bins (nbin), reproducibility threshold (thresh), bin reproducibility (reprd), error rate (ErrBonin) and polymorphy rate (Pol).

| **Colour dye** | **Year of data** | **minbin** | **maxbin** | **rfu** | **thresh** | **nbin** | **BinReprod** | **ErrBonin** | **Pol** |
| --- | --- | --- | --- | --- | --- | --- | --- | --- | --- |
| Blue (FAM) | 2016 | 1.5 | 1.7 | 175 | 75 | 120 | 31.56 | 0.15 | 0.68 |
|  | 2017 | 1.6 | 2.1 | 100 | 75 | 198 | 6.51 | 0.05 | 0.38 |
| Green (HEX) | 2016 | 1.6 | 1.8 | 150 | 75 | 110 | 27.85 | 0.16364 | 0.74 |
|  | 2017 | 1.7 | 2.1 | 150 | 75 | 137 | 9.3 | 0.06 | 0.42 |
| Yellow (NED) | 2016 | 1.7 | 1.8 | 150 | 75 | 105 | 31.67 | 0.15238 | 0.73 |
|  | 2017 | 1.1 | 2.1 | 100 | 75 | 126 | 9.17 | 0.048 | 0.33 |

Table S3. The arcsine transformed percentages of polymorphic epiloci of the 4 groups for 2016 & 2017 treatment years. The data in the current table were used for treatment years’ boxplots and for pairwise comparisons of the same *R. kuepferi* individuals through the two treatment years.

| **Group** | **Year** | **SampleID** | **Externally-methylated** | **Internally-methylated** | **Non-methylated** |
| --- | --- | --- | --- | --- | --- |
| D2 | 2017 | 112_1_2 | 2.73 | 3.23 | 3.47 |
| D2 | 2017 | 115_4_2 | 2.22 | 3.23 | 1.57 |
| D2 | 2017 | 116_1_3 | 1.86 | 2.32 | 2.45 |
| D2 | 2017 | 116_3_1 | 2.86 | 3.46 | 3.32 |
| D2 | 2017 | 117_3_3 | 2.42 | 2.6 | 1.57 |
| D2 | 2017 | 202_3_3 | 1.96 | 2.82 | 2.63 |
| D2 | 2017 | 203_2_2 | 1.96 | 2.82 | 2.45 |
| D2 | 2017 | 23_3_3 | 2.14 | 0 | 1.57 |
| D2 | 2017 | 233_2_3 | 3.25 | 2.92 | 3.61 |
| D2 | 2017 | 24_1_2 | 3.45 | 0 | 1.57 |
| D2 | 2017 | 25_1_3 | 2.22 | 2.72 | 1.95 |
| D2 | 2017 | 26_4_1 | 2.82 | 3.01 | 3.03 |
| D2 | 2017 | 27_2_2 | 2.94 | 1.93 | 1.95 |
| D2 | 2017 | 28_2_3 | 3.14 | 2.47 | 1.95 |
| D2 | 2017 | 28B_1_2 | 2.36 | 3.09 | 3.14 |
| D2 | 2017 | 29_4_2 | 1.48 | 2.47 | 0.98 |
| D2 | 2017 | 3_2_2 | 0.34 | 2.32 | 2.63 |
| D2 | 2017 | 3_3_2 | 0 | 0 | 2.63 |
| D2 | 2017 | 30_1_2 | 2.97 | 2.47 | 1.57 |
| D2 | 2017 | 31_2_3 | 2.9 | 2.32 | 1.95 |
| D2 | 2017 | 31B/32B_2_1 | 1.86 | 2.72 | 1.95 |
| D2 | 2017 | 31B_3_3 | 1.75 | 3.23 | 2.92 |
| D2 | 2017 | 32_3_3 | 2.05 | 2.32 | 0.98 |
| D2 | 2017 | 32B_1_1 | 3.2 | 2.72 | 2.63 |
| D2 | 2017 | 33_4_2 | 2.29 | 3.23 | 0.98 |
| T2 | 2017 | 106_2_3 | 3.38 | 0 | 1.57 |
| T2 | 2017 | 108_3_3 | 3.14 | 0 | 1.57 |
| T2 | 2017 | 111_2_3 | 3.45 | 0 | 1.57 |
| T2 | 2017 | 17_4_2 | 0.34 | 0.76 | 2.45 |
| T2 | 2017 | 36_2_1 | 3.86 | 0.76 | 1.57 |
| T2 | 2017 | 37_4_1 | 3.72 | 0 | 1.57 |
| T2 | 2017 | 40_3_1 | 2.42 | 1.65 | 1.57 |
| T2 | 2017 | 41_2_2 | 1.32 | 1.93 | 1.57 |
| T2 | 2017 | 42_3_2 | 2.29 | 2.6 | 1.57 |
| T2 | 2017 | 45_1_2 | 2.9 | 1.65 | 1.57 |
| T2 | 2017 | 47_2_2 | 3.11 | 1.93 | 1.57 |
| T2 | 2017 | 48_4_2 | 2.64 | 1.93 | 1.57 |
| T2 | 2017 | 50_2_1 | 3.07 | 3.61 | 2.23 |
| T2 | 2017 | 53_3_1 | 2.54 | 1.93 | 4.05 |
| T2 | 2017 | 54_2_2 | 2.78 | 1.29 | 1.57 |
| T2 | 2017 | 55_3_1 | 2.36 | 0.76 | 1.57 |
| T2 | 2017 | 58_1_3 | 2.14 | 2.47 | 1.57 |
| T2 | 2017 | 66_2_2 | 3.11 | 0.76 | 1.57 |
| T2 | 2017 | 79_3_3 | 3.57 | 3.35 | 1.95 |
| T2 | 2017 | 82_3_3 | 2.9 | 0 | 1.57 |
| T2 | 2017 | 83_3_1 | 2.05 | 0 | 1.57 |
| T2 | 2017 | 84_4_2 | 3.25 | 0 | 1.57 |
| T2 | 2017 | 90_2_3 | 3.2 | 0 | 1.57 |
| T2 | 2017 | 96_2_3 | 3.3 | 0.76 | 1.57 |
| D1 | 2017 | 112_3_1 | 3.4 | 2.32 | 2.63 |
| D1 | 2017 | 112_3_2 | 3.2 | 3.09 | 0.98 |
| D1 | 2017 | 115_3_3 | 3.14 | 2.82 | 2.23 |
| D1 | 2017 | 116_1_1 | 2.97 | 2.6 | 1.57 |
| D1 | 2017 | 116_3_3 | 2.14 | 2.32 | 0.98 |
| D1 | 2017 | 117_3_2 | 1.32 | 2.6 | 0.98 |
| D1 | 2017 | 203_4_3 | 2.42 | 2.32 | 1.95 |
| D1 | 2017 | 23_4_1 | 0.34 | 2.32 | 1.95 |
| D1 | 2017 | 233_2_1 | 1.48 | 2.14 | 4.13 |
| D1 | 2017 | 24_4_3 | 1.13 | 1.93 | 1.57 |
| D1 | 2017 | 25_3_2 | 1.96 | 1.93 | 1.57 |
| D1 | 2017 | 26_3_3 | 3.38 | 1.65 | 2.45 |
| D1 | 2017 | 27_2_1 | 3.01 | 2.82 | 1.95 |
| D1 | 2017 | 28_3_1 | 3.55 | 2.32 | 1.57 |
| D1 | 2017 | 28_3_2 | 1.48 | 1.93 | 1.57 |
| D1 | 2017 | 28B_3_1 | 2.05 | 0.76 | 3.88 |
| D1 | 2017 | 29_1_2 | 2.69 | 1.29 | 4.05 |
| D1 | 2017 | 3_2_3 | 2.22 | 2.92 | 2.23 |
| D1 | 2017 | 30_1_3 | 0.65 | 1.65 | 1.57 |
| D1 | 2017 | 31_3_3 | 1.75 | 3.09 | 3.78 |
| D1 | 2017 | 31B/32B_2_2 | 1.62 | 1.65 | 1.57 |
| D1 | 2017 | 31B_1_3 | 1.32 | 1.65 | 1.57 |
| D1 | 2017 | 32_4_2 | 1.13 | 2.47 | 2.23 |
| D1 | 2017 | 32B_1_2 | 1.32 | 2.32 | 1.95 |
| D1 | 2017 | 33_3_3 | 0.34 | 1.65 | 1.57 |
| T1 | 2017 | 103_4_2 | 2.86 | 2.47 | 1.57 |
| T1 | 2017 | 106_4_1 | 2.69 | 0.76 | 1.57 |
| T1 | 2017 | 111_3_2 | 2.9 | 0.76 | 3.78 |
| T1 | 2017 | 111_3_3 | 2.69 | 0 | 1.57 |
| T1 | 2017 | 116_4_1 | 3.01 | 0 | 1.57 |
| T1 | 2017 | 17_4_3 | 3.38 | 2.47 | 2.78 |
| T1 | 2017 | 208_2_1 | 2.22 | 0 | 1.57 |
| T1 | 2017 | 34_2_1 | 3.04 | 2.32 | 2.23 |
| T1 | 2017 | 36_4_1 | 2.69 | 1.65 | 3.83 |
| T1 | 2017 | 37_1_2 | 1.86 | 0.76 | 1.57 |
| T1 | 2017 | 38_1_2 | 3.8 | 2.47 | 1.57 |
| T1 | 2017 | 3B_1_2 | 2.78 | 0 | 1.57 |
| T1 | 2017 | 41_4_3 | 3.81 | 3.01 | 1.57 |
| T1 | 2017 | 42_1_2 | 3.45 | 2.92 | 2.23 |
| T1 | 2017 | 47_3_3 | 2.42 | 1.65 | 1.57 |
| T1 | 2017 | 51_3_1 | 2.64 | 1.65 | 1.57 |
| T1 | 2017 | 53_1_3 | 2.48 | 1.65 | 1.57 |
| T1 | 2017 | 54_2_3 | 1.32 | 1.93 | 1.57 |
| T1 | 2017 | 58_1_1 | 2.73 | 1.65 | 1.95 |
| T1 | 2017 | 58_3_3 | 1.96 | 1.65 | 1.95 |
| T1 | 2017 | 74_1_2 | 2.73 | 1.93 | 1.57 |
| T1 | 2017 | 78_3_2 | 2.42 | 1.93 | 3.92 |
| T1 | 2017 | 79_2_3 | 2.42 | 1.65 | 4.09 |
| T1 | 2017 | 85_4_1 | 2.36 | 1.29 | 1.57 |
| T1 | 2017 | 88_3_1 | 1.75 | 0.76 | 1.57 |
| D1 | 2016 | 112_3_1 | 1.43 | 2.17 | 2.17 |
| D1 | 2016 | 112_3_2 | 2.33 | 3.13 | 3.13 |
| D1 | 2016 | 115_3_3 | 1.97 | 1.24 | 1.24 |
| D1 | 2016 | 116_1_1 | 1.43 | 1.74 | 1.74 |
| D1 | 2016 | 116_3_3 | 2.08 | 2.6 | 2.6 |
| D1 | 2016 | 117_3_2 | 1.59 | 3.02 | 3.02 |
| D1 | 2016 | 203_4_3 | 1.86 | 3.63 | 3.63 |
| D1 | 2016 | 23_4_1 | 1.59 | 3.71 | 3.71 |
| D1 | 2016 | 233_2_1 | 2.25 | 3.94 | 3.94 |
| D1 | 2016 | 24_4_3 | 1.86 | 3.4 | 3.4 |
| D1 | 2016 | 25_3_2 | 1.59 | 3.57 | 3.57 |
| D1 | 2016 | 26_3_3 | 1.73 | 4.23 | 4.23 |
| D1 | 2016 | 27_2_1 | 1.97 | 2.17 | 2.17 |
| D1 | 2016 | 28_3_1 | 1 | 2.9 | 2.9 |
| D1 | 2016 | 28_3_2 | 0.72 | 1.59 | 1.59 |
| D1 | 2016 | 28B_3_1 | 2.33 | 3.5 | 3.5 |
| D1 | 2016 | 29_1_2 | 1.97 | 1.43 | 1.43 |
| D1 | 2016 | 3_2_3 | 2.08 | 4.12 | 4.12 |
| D1 | 2016 | 30_1_3 | 1.59 | 3.45 | 3.45 |
| D1 | 2016 | 31_3_3 | 1.23 | 3.89 | 3.89 |
| D1 | 2016 | 31B/32B_2_2 | 2.65 | 4 | 4 |
| D1 | 2016 | 31B_1_3 | 3.78 | 1.74 | 1.74 |
| D1 | 2016 | 32_4_2 | 1.97 | 3.2 | 3.2 |
| D1 | 2016 | 32B_1_2 | 2.08 | 3.2 | 3.2 |
| D1 | 2016 | 33_3_3 | 1.86 | 3.67 | 3.67 |
| T1 | 2016 | 103_4_2 | 3.87 | 3.2 | 3.2 |
| T1 | 2016 | 106_4_1 | 3.5 | 3.79 | 3.79 |
| T1 | 2016 | 111_3_2 | 3.92 | 2.76 | 2.76 |
| T1 | 2016 | 111_3_3 | 2.76 | 3.73 | 3.73 |
| T1 | 2016 | 116_4_1 | 3.09 | 3.69 | 3.69 |
| T1 | 2016 | 17_4_3 | 1.59 | 4.38 | 4.38 |
| T1 | 2016 | 208_2_1 | 3.22 | 3.9 | 3.9 |
| T1 | 2016 | 34_2_1 | 2.6 | 3.95 | 3.95 |
| T1 | 2016 | 36_4_1 | 3.13 | 3.65 | 3.65 |
| T1 | 2016 | 37_1_2 | 3.59 | 3.73 | 3.73 |
| T1 | 2016 | 38_1_2 | 2.25 | 3.69 | 3.69 |
| T1 | 2016 | 3B_1_2 | 2.65 | 3.61 | 3.61 |
| T1 | 2016 | 41_4_3 | 3.31 | 3.67 | 3.67 |
| T1 | 2016 | 42_1_2 | 3.5 | 3.35 | 3.35 |
| T1 | 2016 | 47_3_3 | 3.4 | 3.59 | 3.59 |
| T1 | 2016 | 51_3_1 | 3.19 | 3.84 | 3.84 |
| T1 | 2016 | 53_1_3 | 3.06 | 3.89 | 3.89 |
| T1 | 2016 | 54_2_3 | 3.54 | 3.59 | 3.59 |
| T1 | 2016 | 58_1_1 | 2.85 | 4.12 | 4.12 |
| T1 | 2016 | 58_3_3 | 3.16 | 3.73 | 3.73 |
| T1 | 2016 | 74_1_2 | 3.87 | 3.2 | 3.2 |
| T1 | 2016 | 78_3_2 | 3.16 | 3.94 | 3.94 |
| T1 | 2016 | 79_2_3 | 2.33 | 3.89 | 3.89 |
| T1 | 2016 | 85_4_1 | 3.22 | 3.61 | 3.61 |
| T1 | 2016 | 88_3_1 | 2.71 | 3.8 | 3.8 |
| D2 | 2016 | 112_1_2 | 1.23 | 1.74 | 1.74 |
| D2 | 2016 | 115_4_2 | 2.81 | 3.37 | 3.37 |
| D2 | 2016 | 116_1_3 | 1.23 | 2.81 | 2.81 |
| D2 | 2016 | 116_3_1 | 1.59 | 3.37 | 3.37 |
| D2 | 2016 | 117_3_3 | 1.73 | 3.57 | 3.57 |
| D2 | 2016 | 202_3_3 | 0.38 | 2.08 | 2.08 |
| D2 | 2016 | 203_2_2 | 2.71 | 3.98 | 3.98 |
| D2 | 2016 | 23_3_3 | 1.86 | 3.69 | 3.69 |
| D2 | 2016 | 233_2_3 | 3.06 | 3.45 | 3.45 |
| D2 | 2016 | 24_1_2 | 2.33 | 3.73 | 3.73 |
| D2 | 2016 | 25_1_3 | 2.47 | 3.61 | 3.61 |
| D2 | 2016 | 26_4_1 | 2.54 | 2.76 | 2.76 |
| D2 | 2016 | 27_2_2 | 3.75 | 2.81 | 2.81 |
| D2 | 2016 | 28_2_3 | 2.47 | 3.87 | 3.87 |
| D2 | 2016 | 28B_1_2 | 2.17 | 4.16 | 4.16 |
| D2 | 2016 | 29_4_2 | 2.4 | 3.52 | 3.52 |
| D2 | 2016 | 3_2_2 | 3.61 | 3.29 | 3.29 |
| D2 | 2016 | 3_3_2 | 3.06 | 3.65 | 3.65 |
| D2 | 2016 | 30_1_2 | 3.16 | 3.35 | 3.35 |
| D2 | 2016 | 31_2_3 | 2.81 | 3.69 | 3.69 |
| D2 | 2016 | 31B_3_3 | 2.17 | 4.23 | 4.23 |
| D2 | 2016 | 32_3_3 | 1.59 | 2.54 | 2.54 |
| D2 | 2016 | 32B/31B_2_1 | 2.54 | 3.65 | 3.65 |
| D2 | 2016 | 32B_1_1 | 2.65 | 3.89 | 3.89 |
| D2 | 2016 | 33_4_2 | 3.19 | 3.71 | 3.71 |
| T2 | 2016 | 106_2_3 | 2.71 | 4.01 | 4.01 |
| T2 | 2016 | 108_3_3 | 2.4 | 4.07 | 4.07 |
| T2 | 2016 | 111_2_3 | 3.56 | 3.1 | 3.1 |
| T2 | 2016 | 17_4_2 | 1.97 | 2.98 | 2.98 |
| T2 | 2016 | 36_2_1 | 3.76 | 3.35 | 3.35 |
| T2 | 2016 | 37_4_1 | 2.9 | 3.82 | 3.82 |
| T2 | 2016 | 40_3_1 | 3.37 | 3.35 | 3.35 |
| T2 | 2016 | 41_2_2 | 3.16 | 3.73 | 3.73 |
| T2 | 2016 | 42_3_2 | 3.42 | 3.32 | 3.32 |
| T2 | 2016 | 45_1_2 | 3.06 | 3.45 | 3.45 |
| T2 | 2016 | 47_2_2 | 3.22 | 3.35 | 3.35 |
| T2 | 2016 | 48_4_2 | 3.5 | 3.61 | 3.61 |
| T2 | 2016 | 50_2_1 | 2.76 | 3.8 | 3.8 |
| T2 | 2016 | 53_3_1 | 2.71 | 3.92 | 3.92 |
| T2 | 2016 | 54_2_2 | 2.81 | 3.75 | 3.75 |
| T2 | 2016 | 55_3_1 | 3.28 | 3.23 | 3.23 |
| T2 | 2016 | 58_1_3 | 2.85 | 3.9 | 3.9 |
| T2 | 2016 | 66_2_2 | 2.6 | 3.73 | 3.73 |
| T2 | 2016 | 79_3_3 | 2.25 | 4.12 | 4.12 |
| T2 | 2016 | 82_3_3 | 3.09 | 3.77 | 3.77 |
| T2 | 2016 | 83_3_1 | 2.65 | 4.19 | 4.19 |
| T2 | 2016 | 84_4_2 | 2.33 | 4.3 | 4.3 |
| T2 | 2016 | 90_2_3 | 3.28 | 3.8 | 3.8 |
| T2 | 2016 | 96_2_3 | 2.47 | 4.19 | 4.19 |
